# The free-living flatworm *Macrostomum lignano*

**DOI:** 10.1186/s13227-020-00150-1

**Published:** 2020-03-02

**Authors:** Jakub Wudarski, Bernhard Egger, Steven A. Ramm, Lukas Schärer, Peter Ladurner, Kira S. Zadesenets, Nikolay B. Rubtsov, Stijn Mouton, Eugene Berezikov

**Affiliations:** 1 European Research Institute for the Biology of Ageing, University of Groningen, University Medical Center Groningen, Antonius Deusinglaan 1, 9713AV Groningen, The Netherlands; 2 Institute of Zoology and Center for Molecular Biosciences Innsbruck, University of Innsbruck, Technikerstr. 25, 6020 Innsbruck, Austria; 3 Department of Evolutionary Biology, Bielefeld University, Morgenbreede 45, 33615 Bielefeld, Germany; 4 Department of Environmental Sciences, Zoological Institute, University of Basel, Vesalgasse 1, 4051 Basel, Switzerland; 5 The Federal Research Center Institute of Cytology and Genetics SB RAS, Prospekt Lavrentyeva 10, Novosibirsk, 630090 Russia

**Keywords:** *Macrostomum*, Flatworms, Regeneration, Neoblasts, Transgenesis, Ageing, Re-diploidization, Sex allocation, Sexual selection, Bio-adhesion

## Abstract

*Macrostomum lignano* is a free-living flatworm that is emerging as an attractive experimental animal for research on a broad range of biological questions. One feature setting it apart from other flatworms is the successful establishment of transgenesis methods, facilitated by a steady supply of eggs in the form of single-cell zygotes that can be readily manipulated. This, in combination with the transparency of the animal and its small size, creates practical advantages for imaging and fluorescence-activated cell sorting in studies related to stem cell biology and regeneration. *M. lignano* can regenerate most of its body parts, including the germline, thanks to the neoblasts, which represent the flatworm stem cell system. Interestingly, neoblasts seem to have a high capacity of cellular maintenance, as *M. lignano* can survive up to 210 Gy of γ-irradiation, and partially offset the negative consequence of ageing. As a non-self-fertilizing simultaneous hermaphrodite that reproduces in a sexual manner, *M. lignano* is also used to study sexual selection and other evolutionary aspects of sexual reproduction. Work over the past several years has led to the development of molecular resources and tools, including high-quality genome and transcriptome assemblies, transcriptional profiling of the germline and somatic neoblasts, gene knockdown, and in situ hybridization. The increasingly detailed characterization of this animal has also resulted in novel research questions, such as bio-adhesion based on its adhesion-release glands and genome evolution due to its recent whole-genome duplication.
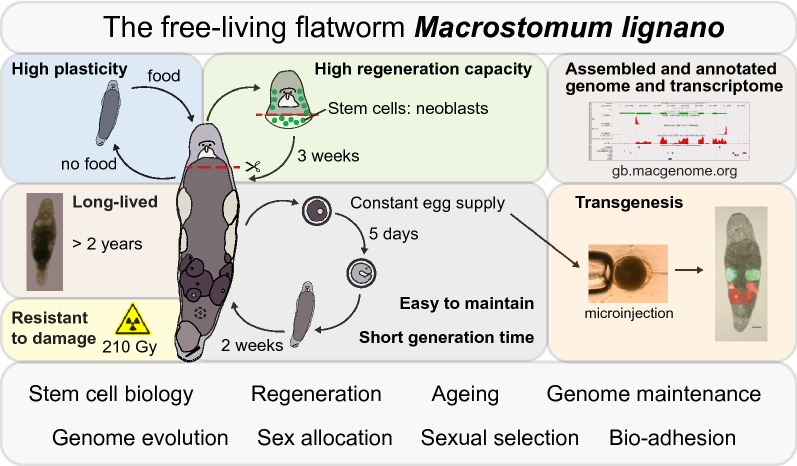

## Natural habitat and life cycle

**Fig. 1:**
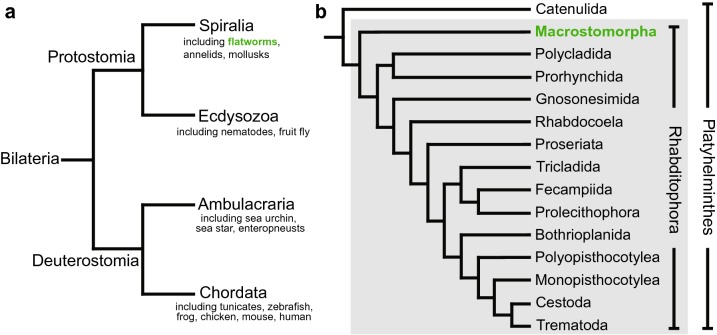
Phylogenetic position of *Macrostomum lignano*. **a** Overview of the systematic position of flatworms in the animal tree of life. **b** Interrelationships of the flatworm orders, modified after [46]. *M. lignano* is a member of the Macrostomorpha (green)

**Fig. 2:**
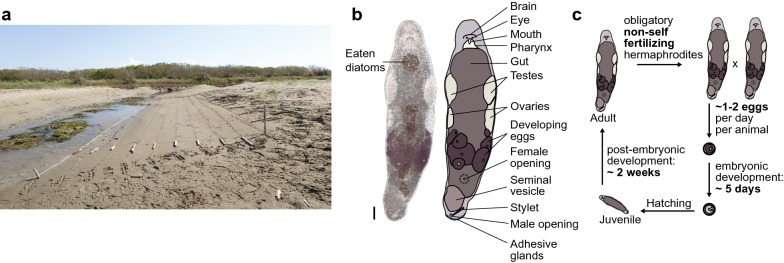
*Macrostomum lignano* habitat, morphology, and life cycle. **a** The natural habitat of *M. lignano* in the upper intertidal zone of the Northern Adriatic. A systematic transect to study the distribution of *Macrostomum* species at Bocca d’Anfora, Italy, is shown. **b** Bright-field image and schematic representation of an adult worm (lightly squeezed to allow for better observation). Scale bar 100 µm. **c** Schematic visualization of the *M. lignano* life cycle

The flatworm *Macrostomum lignano* is a free-living, marine species belonging to the Macrostomorpha, the earliest branching clade of Rhabditophora [1] (Fig. 1). The species is found on the beaches of the Northern Adriatic and the Aegean Sea. It is adapted to live in the interstitial spaces between sand grains in the upper intertidal zone, a zone not covered by water during every tidal cycle (Fig. 2a). As a result, the animals are exposed to variable environmental conditions and can cope with a broad range of temperatures, salinities, and oxygen concentrations [2–4]. Adult animals are 1–2 mm in length and 0.3 mm in width (Fig. 2b). The epidermis is multi-ciliated and animals move by coordinated ciliary beating. Beneath the epidermis and a thin basal matrix lies the body wall musculature consisting of circular, diagonal, and longitudinal fibers, which permit the animal to perform body movements (Additional file 1). Major organ systems include a central nervous system with a brain anterior to the paired eyes, a mouth, a pharynx and a blind-ending rod-shaped gut, paired testes anterior to paired ovaries, female and male genital openings, and a sclerotised copulatory organ in the tail plate [5]. *M. lignano* is an obligately non-self-fertilizing species that reproduces exclusively in a sexual manner [6] (Additional file 2). Worms lay single-cell fertilized eggs (zygotes), and embryonic development takes 5 days (Additional file 3), after which a juvenile emerges through a pre-determined hatch in the egg shell and directly develops into a mature animal in less than 2 weeks (Fig. 2c) [7].

## Field collection and laboratory culture

*Macrostomum lignano* can be readily extracted from field-collected sand samples using a classical meiofauna extraction technique, i.e., decantation after MgCl_2_ anaesthesia. Often, samples contain many meiofaunal species, including multiple species of *Macrostomum*. Species identification, therefore, requires detailed observation of a lightly squeezed worm in a so-called squeeze preparation under a compound microscope. The main distinguishing feature of *M. lignano* compared to other *Macrostomum* species is the shape and size of the male copulatory stylet [5].

*Macrostomum lignano* is a lab-friendly organism. Worms can be kept in Petri dishes with artificial sea water as the medium and unicellular diatom algae of the species *Nitzschia curvilineata* as food source. Starvation causes animals to decrease in size, regress the reproductive system, and reduce mitotic activity of the neoblasts. Feeding such animals will, in turn, induce an increase of neoblast proliferation, growth, and re-establishment of the germline [8]. The optimal laboratory conditions are 20 °C and a 14 h/10 h day/night cycle. However, the worms can survive in a temperature range between 4 °C and 37 °C [2], making it easy to maintain the culture even without specialized equipment. *M. lignano* exhibits negative phototaxis, which helps concentrating the animals at a desired spot in a Petri dish (Additional file 4). An adult animal produces 1–2 eggs per day, and a group of 20 worms can produce over 200 progeny in 1 week, providing ample access to research material. In addition, there are various worm lines available including many inbred (e.g., DV1, NL12) and transgenic (e.g., HUB1 with nearly ubiquitous expression of GFP, NL24 with mScarlet expression in ovaries and mNeonGreen in testes) lines [9, 10].

## Major interests and research questions

### Stem cells and regeneration

**Fig. 3:**
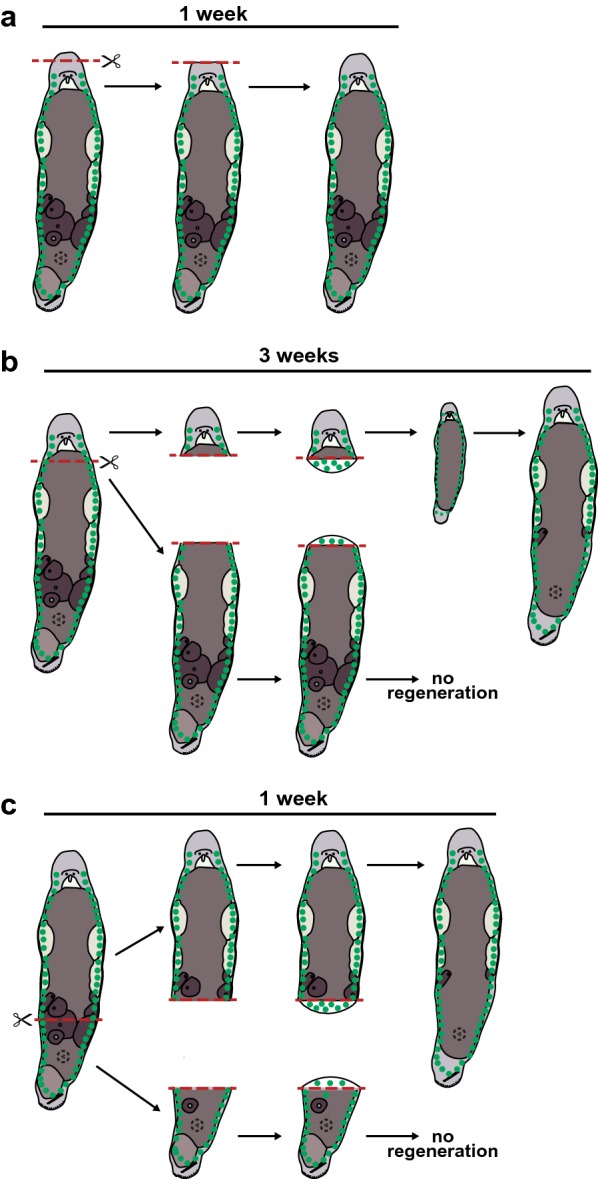
Regeneration capacity of *M. lignano*. In all worms, the green dots represent neoblasts. **a** Anterior regeneration is limited to the rostrum, the region anterior to the eyes and brain. **b**, **c** Posterior regeneration is characterized by the formation of a blastema, an accumulation of proliferating neoblasts, which forms within 48 h after amputation. **b** In case of whole-body amputation, regeneration and remodeling of the remaining tissues of the head will result in a complete but small worm. This worm will then grow into a full adult with new gonads. **c** After amputation of the tail, the missing tissue will be regenerated within a week

*Macrostomum lignano* has a large population of stem cells, called neoblasts, which are located in two lateral bands in the parenchyma, in close proximity to the main lateral nerve cords, and merge in the tail plate [11]. The rostrum, the region anterior of the eyes, is devoid of neoblasts (Fig. 3). Neoblasts are defined by their ability to divide—they are the only proliferating somatic cells in the animal and thus the only source of new cells [11–14]. The neoblast population drives a high cellular turnover during adult tissue homeostasis and provides the regeneration capacity [12]. As in planarians, the neoblast pool of *M. lignano* is assumed to be heterogeneous, including pluripotent and lineage-restricted stem cells. This is supported by indications that a small fraction of the neoblast population is slow cycling or quiescent [13, 15]. Transcriptional profiling of somatic neoblasts and germline cells was recently performed [16]. However, direct evidence for neoblast heterogeneity and pluripotency in *M. lignano* is still lacking, and identifying different types of neoblasts and characterizing the molecular regulation of differentiation into both somatic and germline lineages are a major open research question.

*Macrostomum lignano* can regenerate missing body parts anterior to the brain and posterior to the pharynx (Fig. 3). The regeneration process can be easily observed (Additional file 5), and includes stages of wound closure, formation of a blastema, and the subsequent restoration of the missing tissues and organs [17]. How this complex process is regulated remains poorly understood, and we envision that the experimental power of *M. lignano* will help to unravel the molecular mechanisms behind all stages of regeneration, including initiation, proliferation, and patterning.

### Ageing and resistance to DNA damage

The regeneration capacity is maintained with advancing age, and worms have a lifespan of more than 2 years [18, 19], which is remarkable for an animal of this small size. In contrast to asexual planarians, *M. lignano* does, however, demonstrate phenotypic signs of ageing, such as the appearance of cysts and loss of eyes [18, 19]. Analysis of RNA-sequencing data of ageing animals revealed a significant age-dependent upregulation of neoblast transcripts and several pro-longevity genes, suggesting that *M. lignano* evolved molecular mechanisms to maintain stem cell function and, at least partially, offset the negative consequences of ageing [18]. Dissecting the ageing resilience mechanisms in *M. lignano* may provide significant novel insights for ageing research. One such mechanism is probably connected to genome maintenance, since the worms are highly resistant to external sources of DNA damage, such as ionizing radiation. Full elimination of neoblasts in *M. lignano* requires a fractionated total dose of 210 Gy of γ-irradiation, compared to just a few Gy in mammals [20]. At doses below 210 Gy, the stem cell system can recover and animals survive [16, 20]. Thus, investigation of molecular mechanisms of DNA protection and repair in *M. lignano* holds great promise.

### Genome re-diploidization

**Fig. 4:**
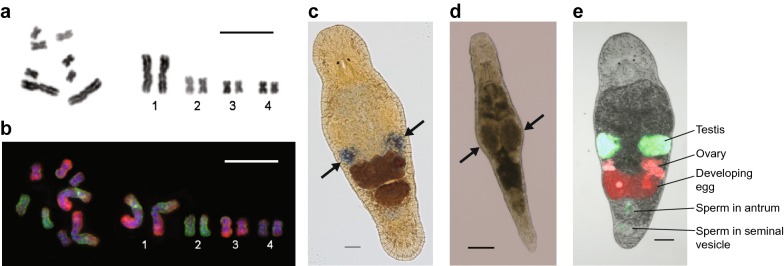
Experimental approaches in *M. lignano.***a** Metaphase chromosomes (left) and karyotype (right) of *M. lignano* (2*n* = 8). Chromosomes were counterstained with DAPI (inverted image). Scale bar 10 µm. **b** Fluorescence in situ hybridization (FISH) with microdissected DNA probes derived from chromosome 2 (*Mli2*, green signal) and distal parts of p- and q-arms of chromosome 1 (*Mli1dist*, red signal) [25]. Scale bar 10 µm. **c** Whole-mount in situ hybridization showing expression of the *CABP7* gene [9] in the ovaries (indicated by arrows). **d** RNAi knockdown phenotype of the *Mlig*-*Sperm1* gene [45] showing enlarged testes (indicated by arrows). **e** Composite image of the NL24 transgenic line [9] showing expression of mNeonGreen under the testis-specific promoter of the *ELAV4* gene, and mScarlet under the ovary-specific promoter of the *CABP7* gene. Note that ELAV4 is expressed in sperm, which is also visible in the seminal vesicle, as well as the female antrum, where it was deposited by another NL24 animal. Scale bars in** c**–**e** are 100 µm

The *M. lignano* haploid genome size is 502 Mb, [9] and its karyotype (2*n* = 8) consists of a pair of large and three pairs of small metacentric chromosomes (Fig. 4a) [21, 22]. Interestingly, in some laboratory lines, including the DV1 line used for the initial genome sequencing [9, 23], chromosome polymorphisms were revealed, associated mostly with copy-number variation of the large chromosome [9, 22], resulting in an increase of the genome size to 742 Mb [9]. Fluorescence in situ hybridization (FISH) analyses suggest that the largest chromosome is derived from a fusion of all small chromosomes, followed by deletions and inversions of some chromosome regions (Fig. 4b), indicating that the modern genome of *M. lignano* may have formed through a recent whole-genome duplication event followed by re-diploidization, including fusion of one full haploid chromosome set into one large metacentric chromosome [24, 25]. This peculiarity of *M. lignano* makes it attractive for studies of early stages of genome and chromosome evolution after whole-genome duplication in animals.

### Embryonic development

*Macrostomum lignano* undergoes a modified spiral-cleavage pattern (Additional file 3), which deviates after the eight-cell stage when yolk-rich and opaque hull cells of embryonic origin start surrounding the inner blastomeres, impeding live observations [7, 26]. This challenge can be overcome with advanced microscopy, such as 4D microscopy [26] and light sheet microscopy [27], or with histological sections [7, 26]. In contrast to other spiralians, the mesentoblast (forming major parts of meso- and endoderm) is not a descendant of blastomere 4d in *M. lignano* [21]. The unusual blastomeric origin of the meso- and endoderm compared to other flatworms (polyclads and prorhynchids) is, therefore, a major research question.

### Evolution of sexual reproduction

*Macrostomum lignano* has long been used as a model system for understanding evolutionary aspects of sex allocation, sexual selection, and sexual conflict in simultaneous hermaphrodites. This is aided by the transparency of the worm permitting in vivo observations of internal structures and processes, including the non-invasive measurement of gonad size and the tracking of GFP-expressing sperm [28, 29]. The species provides some of the strongest empirical support for sex allocation theory pertaining to how individuals should optimally partition investment between their male and female sex functions [6, 28, 30, 31]. Moreover, the genus *Macrostomum* exhibits diversity in reproductive traits, including in behaviour and morphology, making it well suited also for comparative studies of reproductive trait evolution [32]. A major research question now is to better understand how *Macrostomum* species with contrasting and divergent reproductive behaviours and morphologies differ with respect to the mechanisms of sexual selection and sexual conflict.

### Bio-adhesion

*Macrostomum lignano* can attach to any natural substrate, followed by controlled detachment [33, 34]. This reversible attachment relies on a duo-gland adhesion-release system which consists of ~ 130 adhesive organs located on the ventral side of the tail plate [33, 35]. Each adhesive organ is comprised of three cell types: an adhesive gland cell, a releasing gland cell, and a modified epidermal cell called the anchor cell. Recent studies revealed a number of adhesion-related transcripts and two large proteins that mediate temporary adhesion of *M. lignano* [34, 36]. Using proteomic data, antibody, and lectin staining, as well as interference of attachment using specific molecules and surfaces, a model for *M. lignano* attachment and release was proposed [34]. Overall, a better understanding of *M. lignano* bio-adhesion could lead to the generation of synthetic equivalents for medical and industrial applications.

## Experimental approaches

### Immunohistochemistry and in situ hybridization

The small, transparent body and detailed morphological understanding facilitate the use of light and fluorescent imaging in whole animals. Antibody labeling is commonly used to label proliferating neoblasts by means of BrdU incorporation and a polyclonal antibody against phosphorylated Histone H3 [11]. Polyclonal antibodies against, e.g., Vasa [33], Piwi [37], and Boule [38], were produced. In addition, monoclonal antibodies against various cell types, tissues and organs (e.g., spermatids, ventral nerve cord, prostate glands, gut, muscles, and epidermis) were developed [39]. Patterns of gene expression can be visualized in whole animals by in situ hybridization (Fig. 4c) [40].

### RNAi

RNA interference (RNAi) is performed by simply soaking animals in double-stranded RNA (dsRNA) dissolved in culture medium (Fig. 4d). While this technique is easy, it is labor-intensive and has a rather low throughput. The dsRNA fragments can be made in the laboratory with an in vitro transcription protocol [41].

### Transgenesis

The key feature making *M. lignano* stands out among other flatworm model organisms is the availability of transgenic techniques. The worms lay large, single-cell, fertilized eggs that can be microinjected using standard micromanipulation equipment. An established microinjection protocol, coupled with high-quality genome and transcriptome assemblies and the short generation time of the animal, enables the creation of stable transgenic lines in a matter of a few weeks. Several lines with tissue-specific and heatshock-inducible expression are already available (Fig. 4e, Additional file 6) [2, 9]. The published transgenesis approach relies on random integration of injected DNA constructs into the genome and does not require specialized vectors. Stable transgenic lines can be obtained from 1–8% of the injected eggs [9], and around 30–50 eggs can typically be injected in a single session. The use of other transgenesis methods, such as transposon-mediated integration, homologous recombination, and the CRISPR/Cas9 system for genome editing, should be feasible in *M. lignano,* but remain to be developed.

### Live imaging

The transparency of *M. lignano* is especially advantageous for live imaging (Additional file 7). Worms can be immobilized using MgCl_2_ as an anaesthetic and squeeze preparations. The small thickness of the body makes it easy to observe changes in worm morphology even under low magnification stereoscopes and is very helpful for confocal imaging.

### Flow cytometry and FACS

Worms can be macerated into a single-cell suspension using Otto buffers [12] or Accutase. Subsequent flow cytometry and FACS can be performed using standard techniques.

### Karyotyping

A single-worm karyotyping technique allows monitoring of karyotypes in laboratory cultures of worms (Fig. 4a) [22]. The detailed chromosome organization can be investigated by FISH using different types of DNA probes, including microdissected region- and chromosome-specific DNA probes (Fig. 4b) [24, 25]. Since *M. lignano* shows chromosomal polymorphisms [9, 22], it is important to regularly monitor laboratory cultures for the spontaneous duplication of the large chromosome, which can be performed using flow cytometry [9]. Although the chromosomal polymorphisms potentially complicate the use of *M. lignano* as a model for genetic studies, in practice, we have not experienced such problems when using an inbred line NL12, which is derived from wild-type line NL10 [9] and shows a stable karyotype (2*n* = 8).

### Behaviour

The small size of the worms, in combination with digital time-lapse video recording, permits very efficient observation of mating interactions of many individuals simultaneously (Additional file 8), facilitating detailed and well-replicated behavioural studies [29, 42–44]. Since it is easy to generate a lot of behavioural observations, it would be interesting to take advantage of recent developments in image analysis and machine learning, to convert such observations into quantitative data in an automated and time-efficient way.

## Research community and resources

### Macrostomum meeting, ISFB

The *Macrostomum* research community currently comprises about ten laboratories worldwide. *Macrostomum* researchers are meeting approximately yearly since 2007 in the format of the 2-day International *Macrostomum* Meeting (IMM), which all researchers interested in initiating research on *Macrostomum* are highly welcome to join. Moreover, many members of the *Macrostomum* research community also regularly attend the International Symposium on Flatworm Biology (ISFB), which usually takes place every 3 years and brings together researchers that work on free-living flatworms, parasitic flatworms, and acoels.

### The Macrostomorpha Taxonomy and Phylogeny website

A good reference for field collection, observation, and documentation of *Macrostomum* is the Methods section of the Macrostomorpha Taxonomy and Phylogeny website (http://macrostomorpha.info). This website contains digital versions of most of the taxonomic publications in the genus *Macrostomum* and its parent taxon Macrostomorpha, and serves as a repository for information about taxonomic-type specimens and images of reference specimens (digital hologenophore vouchers) that have been used in molecular phylogenetic analyses in this group of flatworms [32].

### Genome, transcriptome, genome browser

The most recent annotated *M. lignano* genome assembly version Mlig_3_7_DV1 [9] is available at GenBank (acc. no. NIVC00000000.1) and on the *Macrostomum* genome resources website (http://www.macgenome.org). The genome can be explored using the UCSC genome browser interface at http://gb.macgenome.org.

### Nanotomy

A whole-animal electron microscopy atlas obtained at the nanoscale provides a “Google-Earth” style of data presentation and navigation at different levels of resolution [45], and is accessible at http://www.nanotomy.org/OA/Macrostomum.

### Neoblast and ageing data interfaces

There are two web interfaces, http://neoblast.macgenome.org and http://ageing.macgenome.org, which provide a straightforward way to search, visualize, and analyse gene expression data generated in the recent *M. lignano* neoblast/germline and ageing studies [16, 18].

## Supplementary information

**Additional file 1.** Movie of adult *M. lignano* animals eating diatom algae.

**Additional file 2.** Movie of mating worms. Two individuals of *M. lignano* are engaged in a reciprocal mating, which is followed by one individual performing the post-copulatory suck behavior (from 22–27 s), after which some sperm can be seen sticking out of the vagina of the worm (final frames). Video from https://www.flickr.com/photos/lukas_scharer/15281560757 under a CC-BY 2.0 licence.

**Additional file 3.** Movie of the early stages of embryonic development in *M. lignano*.

**Additional file 4.** Movie demonstrating negative phototaxis behaviour in *M. lignano*.

**Additional file 5.** Movie of an amputated *M. lignano* head. It will regenerate into a full animal within 3 weeks.

**Additional file 6.** Movie of the transgenic *M. lignano* line NL24, where testes are marked by expression of mNeonGreen under the ELAV4 promoter and ovaries by expression of mScarlet under the CABP7 promoter.

**Additional file 7.** Movie showing live imaging of the *M. lignano* tail region. The high level of transparency of these worms permits detailed observations of anatomical structures in living worms, namely, in order of appearance in the video, the adhesive glands (dotted arc), the male genital opening (small ciliated circle), the large drop-shaped and sperm-filled false seminal vesicle (on the right), the smaller and muscular true seminal vesicle, the vesicula granulorum (with prostate gland granules), the copulatory stylet (long tube from left to right), and some rotating food particles inside of the lumen of the gut.

**Additional file 8.** Observations on the mating interactions in many pairs of *M. lignano*. A 12 s clip of a longer time-lapse video (captured at 1 frame per second and played back at 10 frames per second) showing the interactions in a total of 18 pairs, each placed into individual 4 µl drops in an observation chamber. Within many of the pairs one can observe precopulatory, copulatory, and postcopulatory interactions.

## Data Availability

Not applicable.
